# Circular RNAs and their associations with breast cancer subtypes

**DOI:** 10.18632/oncotarget.13134

**Published:** 2016-11-05

**Authors:** Asha A. Nair, Nifang Niu, Xiaojia Tang, Kevin J. Thompson, Liewei Wang, Jean-Pierre Kocher, Subbaya Subramanian, Krishna R. Kalari

**Affiliations:** ^1^ Department of Health Sciences Research, Mayo Clinic, Rochester, MN, USA; ^2^ Division of Genomic and Molecular Pathology, University of Chicago, Chicago, IL, USA; ^3^ Department of Pharmacology, Mayo Clinic, Rochester, MN, USA; ^4^ Division of Basic and Translational Research, University of Minnesota, Minneapolis, MN, USA

**Keywords:** circular RNA, circ-seq, breast cancer, molecular subtypes, proliferation

## Abstract

Circular RNAs (circRNAs) are highly stable forms of non-coding RNAs with diverse biological functions. They are implicated in modulation of gene expression thus affecting various cellular and disease processes. Based on existing bioinformatics approaches, we developed a comprehensive workflow called Circ-Seq to identify and report expressed circRNAs. Circ-Seq also provides informative genomic annotation along circRNA fused junctions thus allowing prioritization of circRNA candidates. We applied Circ-Seq first to RNA-sequence data from breast cancer cell lines and validated one of the large circRNAs identified. Circ-Seq was then applied to a larger cohort of breast cancer samples (*n* = 885) provided by The Cancer Genome Atlas (TCGA), including tumors and normal-adjacent tissue samples. Notably, circRNA results reveal that normal-adjacent tissues in estrogen receptor positive (ER+) subtype have relatively higher numbers of circRNAs than tumor samples in TCGA. Similar phenomenon of high circRNA numbers were observed in normal breast-mammary tissues from the Genotype-Tissue Expression (GTEx) project. Finally, we observed that number of circRNAs in normal-adjacent samples of ER+ subtype is inversely correlated to the risk-of-relapse proliferation (ROR-P) score for proliferating genes, suggesting that circRNA frequency may be a marker for cell proliferation in breast cancer. The Circ-Seq workflow will function for both single and multi-threaded compute environments. We believe that Circ-Seq will be a valuable tool to identify circRNAs useful in the diagnosis and treatment of other cancers and complex diseases.

## INTRODUCTION

Circular RNAs (circRNAs) are recently discovered members of noncoding RNAs. They range in length from a few hundred to thousands of nucleotides [1]. In contrast to linear RNA transcripts, which are normally spliced tail-to-head, circRNAs are formed by the covalent bonding of their 3′ and 5′ (head-to-tail) ends [2]. The lack of open sites at the 5′ and 3′ ends exempts circRNAs from endonuclease degradation [3], making them stable in cells [4]. When circRNAs were initially identified in plants, they were considered pathogenic because of their structural similarity to viruses [5, 6]. Meanwhile, circRNAs observed in mammalian cells around the same time were thought to result from splicing errors [7–9]. However, more recent studies of circRNAs in drosophila, mouse, and other eukaryotes suggest that these RNA molecules are evolutionarily conserved and thus are not simple artifacts of faulty splicing [10, 11]. In addition, advances in sequencing technology and bioinformatics analyses have renewed interest in these forms of RNA transcripts [2, 12, 13].

After discovering that circRNAs are highly abundant in not only *C. elegans* and zebrafish, but also mouse and human, researchers have begun to uncover many intriguing facets of these diverse RNAs [3]. Many studies have confirmed that circRNAs possess significant pre- and post-transcriptional regulatory functions in mammalian cells [1, 13, 14] and changes in the abundance of circRNAs can adversely affect gene expression [15, 16]. Recent studies indicate that some of the most common functions of circRNAs include their active participation in pre-mRNA splicing [10] as well as promoting transcription of their parent mRNAs [17]. Apart from the above, circRNAs can sometimes serve as microRNA sponges, such as the human circRNA CDR1as, which was shown to contain over 70 binding sites for miR-7 [12, 18].

Stable, cell-free circRNAs have been found in saliva [19] and exosomes [20], making them promising candidates for diagnosis and therapeutics. In particular, discovering disease-specific circRNAs could help identify diagnostic targets in heterogeneous diseases such as cancer. Memczak *et al.* and Salzman *et al.* have developed bioinformatics approaches to detect circRNAs using high-throughput transcriptome sequencing, and to date, several hundred human circRNAs have been identified and cataloged [2, 12, 21]. However, the significance of these RNAs in health and disease is still poorly understood. Recently, Bachmayr-Heyda *et al.* reported that colorectal tumor samples have lower number of circRNAs compared to matched normal colon mucosa [22]. It is known that circRNAs are also associated with single nucleotide polymorphisms linked to a wide range of diseases, including various types of cancer, Parkinson's disease, Alzheimer's disease, multiple sclerosis, and schizophrenia [23].

Here, we have enhanced existing methodologies of circRNA detection [12] and developed a parallelized and configurable workflow, Circ-Seq, that annotates and reports expressed and exclusive circRNAs as final candidates from the analysis. We applied Circ-Seq to one of the largest transcriptome sequencing data available for breast cancer samples, provided by The Cancer Genome Atlas (TCGA) consortium. We identified unique and novel circRNAs present in breast tumor samples and normal-adjacent breast tissue. We identified circRNAs specific to breast tumor samples and catalogued circRNAs unique to each of the three breast cancer subtypes: triple negative (TN), estrogen receptor positive (ER+), and ErbB2 overexpressed–HER2 positive (HER2+). Notably, a lower number of circRNAs were observed in breast tumors compared to both normal-adjacent breast tissue from TCGA as well as normal mammary tissue samples from GTEx. Finally, using a panel of 11 cell proliferation gene markers (ROR-P score), we show that the number of circRNAs detected in ER+ tumor is associated with gene proliferation markers [24]. We also demonstrate that Luminal B tumors have a distinct trend compared to Luminal A tumors based on their circRNA numbers. On the basis of its ability to detect circRNAs in breast cancer samples, we believe that Circ-Seq will be a valuable tool for researchers to identify circRNAs for diagnosis and treatment of complex diseases.

## RESULTS

### Circ-Seq: an automated workflow for circRNA identification

Using existing bioinformatics approaches for circRNA identification by Memczak *et al.* [12], we developed an integrated analytical workflow called Circ-Seq, for identifying and characterizing circRNAs using high-throughput transcriptome sequencing data. Briefly, it improves the existing methodology by applying filters namely, expression, genomic size and validation filters, to report a more confident final catalog of expressed candidate circRNAs. The expression filter retains circRNAs based on the desired number of junction-spanning reads, which is configurable based on sequencing throughput of the sample being analyzed. Next the genomic size filter is applied to discard any circRNA candidate with tail-to-head genomic distance less than 6 bases. Finally, the validation filter uses BLAT [25] to query circRNAs to ensure they do not represent repetitive regions of the genome. Towards the end of the workflow, circRNA fused junctions of the final candidates are annotated with valuable genomic information. Annotation of whether the circRNA is a spliced product of a single gene (‘intra-gene’) or formed across 2 or more genes (‘inter-gene’), and exon location of its 3′ and 5′ ends (‘exon-exon boundary’ or ‘within_exon’) are provided for users discretion to prioritize circRNA candidates in the final report. The workflow is fully automated and designed to run in a multi-threaded cluster environment and can also be used to analyze single-end or paired-end transcriptome samples. Circ-Seq workflow can be downloaded from (http://bioinformaticstools.mayo.edu/research/circ-seq/).

### Identification of circRNAs in breast cancer cell lines

To demonstrate the utility of Circ-Seq, we first tested the workflow on the transcriptomes of eight cell lines, seven from breast tumors (BT20, BT474, MCF7, MDAMB231, MDAMB468, T47D, and ZR751) and one from non-tumor breast cell line (MCF10A) [26], and validated one of the largest circRNA candidates reported by the workflow.

CircRNAs were expressed in both the tumor and normal breast cell lines. As shown in Table 1, we identified an average of 10 circRNAs in the triple negative (TN) cancer cell lines, 22 in the estrogen receptor positive (ER+) cancer cell lines, and 9 in the non-tumor MCF10A cell line. On average, circRNAs detected in the cancer cell lines had 12 junction supporting reads in both TN and ER+ subtypes. Assuming that the exon-intron structures of circRNAs remain intact [17], we observed variable genomic sizes for circRNAs in the tumor and non-tumor cell lines. While the smallest circRNA was of size 51 bases in tumor (ZR751 and BT474) and 70 bases in the non-tumor MCF10A, large circRNAs with genomic sizes exceeding 5kb were found in MCF7, BT474, ZR751 and MDAMB231 tumor lines. After annotating the head-to-tail fused junctions of these circRNAs with gene models, we found that 31% of circRNAs are spliced products of a single gene (intra-gene) and 12% are inter-gene circRNAs. Additionally, 25% of the circRNAs have their head-to-tail fused junctions along legitimate exon-exon boundaries whereas 18% were found with circRNA junctions inside exons and not on the exon boundaries. All circRNAs identified in eight cell lines including the annotations (genomic location, number of supporting reads, inter and intra-gene, and exon boundary annotations) are provided in Supplementary File S1.

**Table 1:** Number of circRNAs identified in breast cell lines using the Circ-Seq workflow

Cell line	Tissue	Breast Cancer Subtype	Total number of circRNAs identified	Final number of circRNAs (after three filters)	Average number of circRNA junction supporting reads
MDAMB231	Tumor	TN	1,111	10	11.2
MDAMB468	Tumor	TN	2,540	15	9.8
BT20	Tumor	TN	1,592	6	15
BT474	Tumor	ER+	4,662	43	14.5
ZR751	Tumor	ER+	3,148	31	11.1
T47D	Tumor	ER+	1,306	5	13.2
MCF7	Tumor	ER+	1,838	9	10
MCF10A	Non-Tumor	–	1,363	9	10.4

### Validation of circRNA in MCF7 breast cancer cells

**Figure 1:**
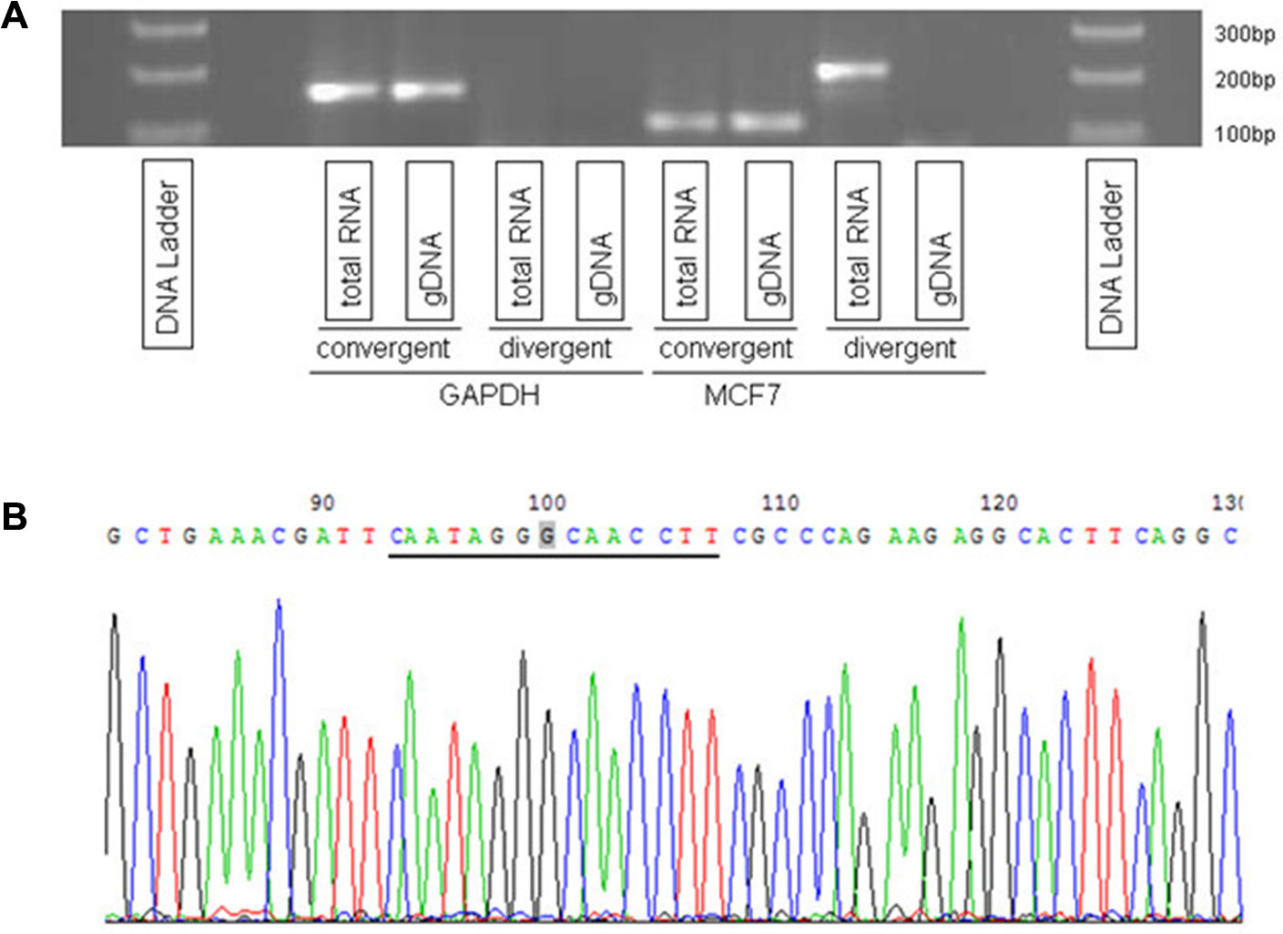
Validation of a circRNA at locus chr14:102,466,325–102,500,789 (**A**) circRNA was amplified by divergent primers using total RNA but not genomic DNA (gDNA). GAPDH was used as a control. (**B**) Head-to-tail splicing was confirmed by Sanger Sequencing.

To establish the reliability of circRNA candidates reported by Circ-Seq, we validated one of the largest circRNAs identified in MCF7, the most widely accessible tumor breast cell line that was available in-house. Circ-Seq results for MCF7 indicated that 2 out of 9 circRNAs were found to span legitimate exon-exon boundaries, of which one had a genomic size of 64 bases and the other 7 kb (see Supplementary File S1 for details). Since some circRNAs were previously reported to act as microRNA sponges and thus had to be long enough to harbor multiple microRNA binding sites [18], we decided to select the largest out of the 2 circRNAs in MCF7 for validation. This circRNA was found at chr14:102,466,325–102,500,789 and had 12 supporting junction-spanning reads. The validation consisted of using two independent sets of qRT-PCR experiments. In order to validate the existence of circRNAs, two different primers were prepared – convergent and divergent [12]. Convergent primers are traditional primers that confirm existence of linear or tail-to-head (5′ to 3′) RNA transcripts, however divergent primers are designed in a circular or head-to-tail fashion (3′ to 5′) to enable binding to circRNA fragments for validation. As shown in Figure 1A, the divergent primer amplified circRNA from MCF7 total RNA but not from genomic DNA (gDNA) whereas GAPDH, which was used as control, had no results from divergent primers but confirmed its linear RNA using convergent primers. Additionally, Sanger sequencing of the qRT-PCR product validated the head-to-tail splicing. In Figure 1B, the underlined genomic sequence CAATAGGGCAACCTT represents the circRNA spliced junction with the 3′ tail fusing to 5′ head at the highlighted ‘G’ nucleotide.

### Presence of circRNAs in TCGA breast cancer transcriptomes

We applied Circ-Seq workflow to 885 whole-transcriptome sequences from breast tumor and normal-adjacent samples provided by the TCGA consortium. Our goal was to use this unique repository to identify circRNAs that differ between normal and tumor tissue. CircRNA results from the workflow for 885 RNA-Seq breast TCGA samples are available for download at https://noncodingrnaexplorer.wordpress.com.

### Breast cancer subtype analysis

### circRNAs in tumors and normal-adjacent tissue

Using the Circ-Seq workflow, we processed 128 tumor and 13 normal-adjacent TN samples, 503 tumor and 56 normal-adjacent ER+ samples, and 162 tumor and 20 normal-adjacent HER2+ samples. As shown in Table 2, we observed a total number of 4,542 and 342 circRNAs in tumor and normal-adjacent samples respectively for the TN subtype. Next, we found the number of unique circRNAs that represented exclusive genomic coordinates in tumor and normal-adjacent samples. Note that a unique circRNA is counted once although it may occur in 2 or more samples with the same genomic coordinate. We observed 1,395 unique circRNAs in TN tumor samples and 208 circRNAs in normal-adjacent tissue samples. Similarly, we identified 14,113 (total) and 3,012 (unique) circRNAs in ER+ tumor samples and 2,317 (total) and 1,409 (unique) circRNAs in normal-adjacent tissue samples. Finally, 6,340 (total) and 2,660 (unique) circRNAs were identified in HER2+ tumors and 532 (total) and 284 (unique) in normal-adjacent tissue samples. A summary of the unique circRNAs for the three breast cancer subtypes are shown in Table 2. Detailed information on the genomic coordinates, number of supporting reads and gene and exon annotations for these circRNAs are provided in Supplementary Files S2, S3 and S4 for ER+, TN and HER2+ subtypes respectively.

**Table 2:** Summary of breast tumor, adjacent tissue, and tumor-specific circRNAs in sequence data made available by the cancer genome atlas

	Triple Negative (TN)		Estrogen Receptor (ER+)		ERBB2 overexpressed (HER2+)	
Categories	Tumor	Adjacent	Tumor	Adjacent	Tumor	Adjacent
Total number of samples	128	13	503	56	162	20
Total number of circRNAs	4,542	342	14,113	2,317	6,340	532
Total number of unique circRNAs	1,395	208	3,012	1,409	2,660	284
Ratio of total circRNAs to samples	35	26	28	41	39	27
Ratio of unique circRNAs to samples	12	16	7	25	17	14
Number of unique circRNAs seen in 10% or more samples	729	162	1,086	455	896	193
Number of tumor-specific circRNAs	256	–	288	–	411	–

We further investigated the unique circRNAs between tumor and normal-adjacent to find circRNAs distinct to tumor. We observed that normal circRNAs spanned larger genomic regions (from 3′ head to 5′ tail). Interestingly, within the same genomic region of the normal circRNAs, we found one or more smaller circRNAs that belonged to the tumor samples. Assuming that circRNAs coming from the same region have similar functional implications during transcriptional regulation, we considered such circRNAs as common candidates between tumor and normal-adjacent tissues. Therefore, if a circRNA was identified in tumor and not in the normal-adjacent tissue, we termed such candidates as tumor-specific circRNAs and found 256, 288 and 411 tumor-specific circRNAs in TN, ER+ and HER2+ breast cancer subtypes respectively.

Because the number of normal-adjacent samples *was much smaller* than the number of breast tumor samples (most tumor samples did not have a paired normal-adjacent tissue sample), we also calculated the ratio of unique circRNAs to the number of samples. Interestingly, after normalization, we see that circRNAs have a higher count in normal-adjacent samples, as shown in Table 2. We tested the significance of this observation using ANOVA and found that normal-adjacent samples of ER+ subtype had *p*-value < 8.96e–06 compared to tumor. However, for TN and HER2+ subtypes the probability measure was insignificant (*p*-value > 0.05), and combining all subtypes together also did not show a significant increase in number of normal-adjacent tissue circRNAs (*p*-value 0.11).

### Tumor-specific circRNAs in breast cancer cell lines also present in breast cancer tissues

**Figure 2:**
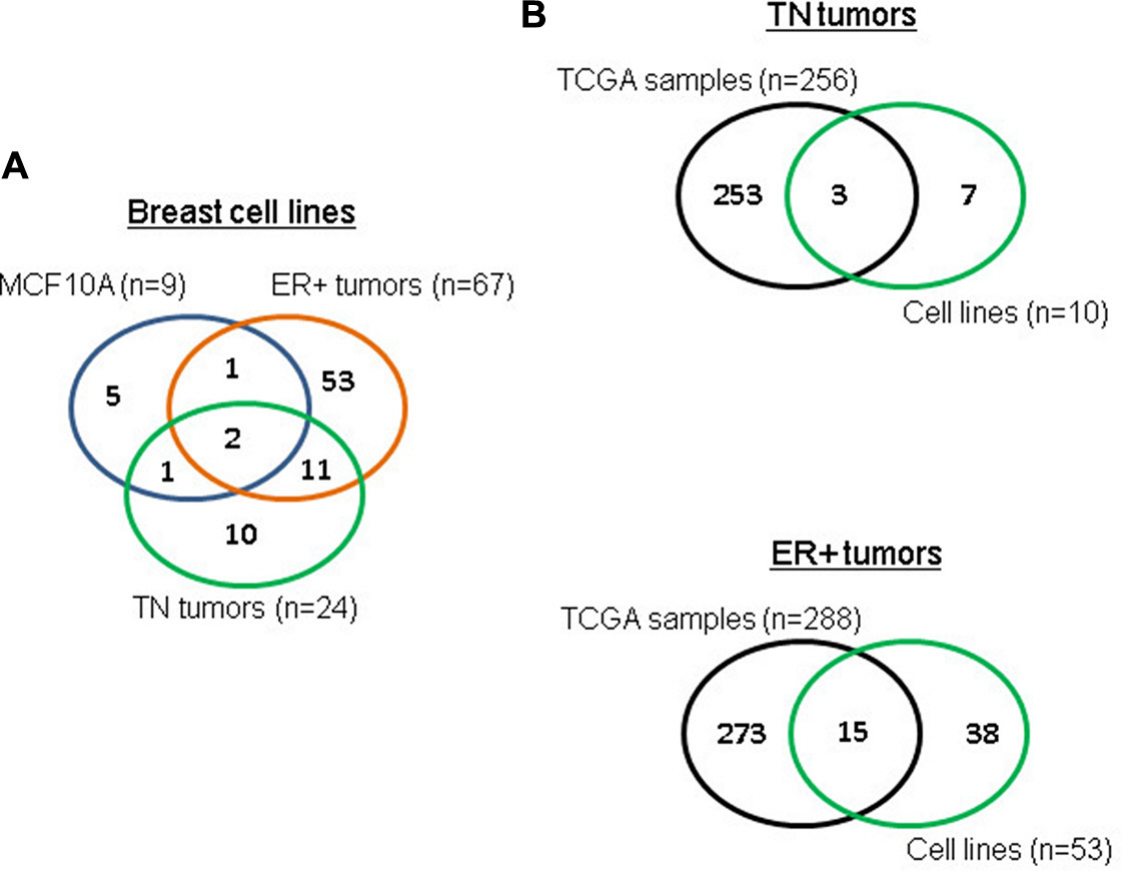
TCGA tumor-specific circRNAs also found in breast cell lines (**A**) overlap of circRNAs between different subtypes for breast cell lines, (**B**) overlap of TN and ER+ tumor-specific circRNAs between TCGA and cell lines.

circRNAs from the TN and ER+ cancer cell lines were compared to those from the non-tumor MCF10A breast cell line (see Table 1 for subtype classification of cell lines; no HER2+ cell lines were available). This comparison yielded 10 TN-specific and 53 ER+ –specific circRNAs (Figure 2A). We checked for common tumor-specific circRNAs between breast cancer cell lines and breast cancer TCGA samples. We also compared these circRNAs to the 256 circRNAs identified earlier in TCGA TN breast cancer samples and the 288 circRNAs obtained from TCGA ER+ breast cancer samples. As shown in Figure 2B, we found that 3 circRNAs were shared between TN breast cancer cell lines and TCGA TN breast cancer samples, and 15 circRNAs were shared between ER+ breast cancer cell lines and TCGA ER+ breast cancer samples.

### Tumor-specific circRNAs are associated with cancer-related canonical pathways

The TN, ER+, and HER2+ breast cancer subtypes have unique prognostic and therapeutic characteristics. Although the gene expression profiles of these subtypes are markedly different [27, 28], a shared population of genes behaves similarly across them. We observed a comparable trend for circRNAs. We found that 42 tumor-specific circRNAs were common across TN, ER+ and HER2+ subtypes. At the same time, we also observed 142 TN, 164 ER+ and 245 HER2+ tumor-specific circRNAs that are exclusive to each subtype.

**Figure 3:**
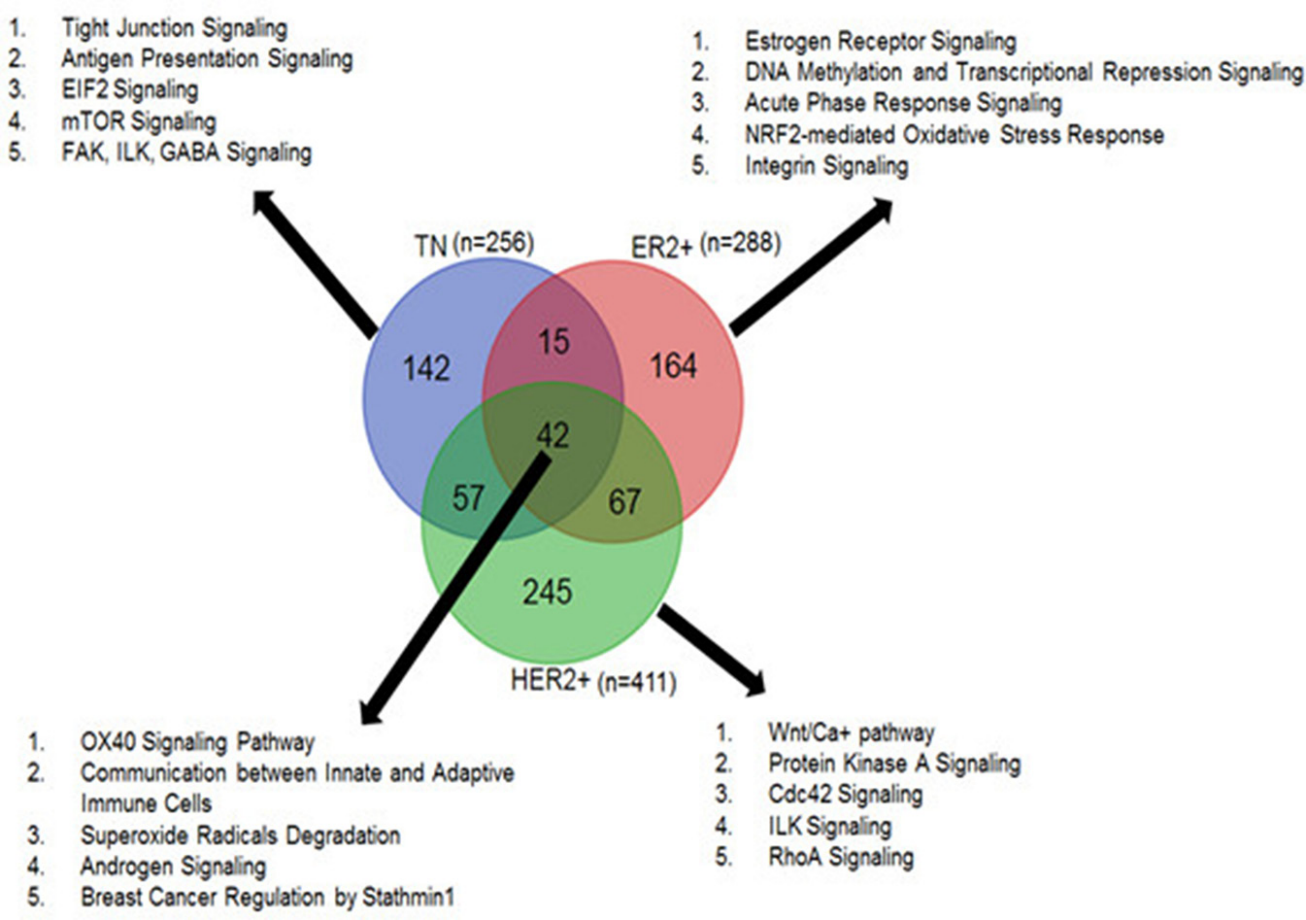
Tumor-specific circRNAs common and unique to TN, ER+ and HER2+ subtypes and the top canonical pathways associated with each subtype

Because circRNAs have post-transcriptional regulatory functions and tend to influence overlapping or neighboring genes [7, 12], we annotated the tumor-specific circRNAs with protein-coding genes using the Ensembl reference system (version GRCh37.75). Pathway analysis demonstrated that most tumor-specific circRNAs were associated with cancer-related canonical pathways. The 42 circRNAs common to all three breast cancer subtypes were annotated with 45 genes, of which 33 genes (*p*-value = 8.43E-05–4.09E-03) were associated with cancer-related pathways. As shown in Figure 3, these circRNAs are likely involved in various hormone signaling, immune cell communication, and OX40 signaling pathways. The circRNAs (*n* = 142) unique to TN tumor samples were linked to a total of 370 genes of which 220 genes (*p*-value = 7.79E-06–1.26E-02) were associated with cancer pathways such as tight junction, antigen presentation, and mTOR signaling pathways. Likewise, HER2+-specific circRNAs (*n* = 245), annotated with over 1,500 protein-coding genes, had 855 cancer-related genes (*p*-value = 1.65E-14–2.24E-03) involved in Wnt signaling, Cdc42, and ILK signaling pathways. The ER+-specific circRNAs (*n* = 164) were found to overlap and/or neighbor 170 genes of which 129 cancer related genes (*p*-value = 2.28E-12–6.82E-03) were associated with estrogen receptor signaling, epigenetic signaling, and oxidative stress response pathways. Pathway analysis results from open source toolkit WebGestalt [29] is also provided in Supplementary File S7.

### Paired normal-adjacent tissue analysis

### Normal-adjacent samples have more unique circRNAs than tumor samples in ER+ subtype

We obtained paired breast tumor and normal-adjacent data from TCGA for 13 TN, 56 ER+, and 20 HER2+ samples. The circRNA results showed that the normal-adjacent samples had a higher number of unique circRNAs than the matched tumors in 5/13 TN patients, 23/56 ER+ and 6/20 HER2+ samples. Using standard paired-*t*-test, again we found that in ER+ cancer, number of circRNAs was higher in normal-adjacent that tumor with *p*-value < 0.027. No correlation was observed between number of unmapped reads and circRNA number (R^2^ = 0.099) and after normalizing for unmapped reads, we still observed significant difference (*p*-value < 0.041) between ER+ normal-adjacent tissue and tumor samples The TN and HER2+ patients did not show significance, *p*-value > 0.05 (Supplementary Figure S1) and combining all subtypes (89 pairs) yielded *p*-value < 0.1. Supplementary File S5 lists all breast cancer TCGA paired samples with the number of unique circRNAs observed in their normal-adjacent and tumor tissues.

### Large number of circRNAs observed in normal breast samples from Gtex

**Figure 4:**
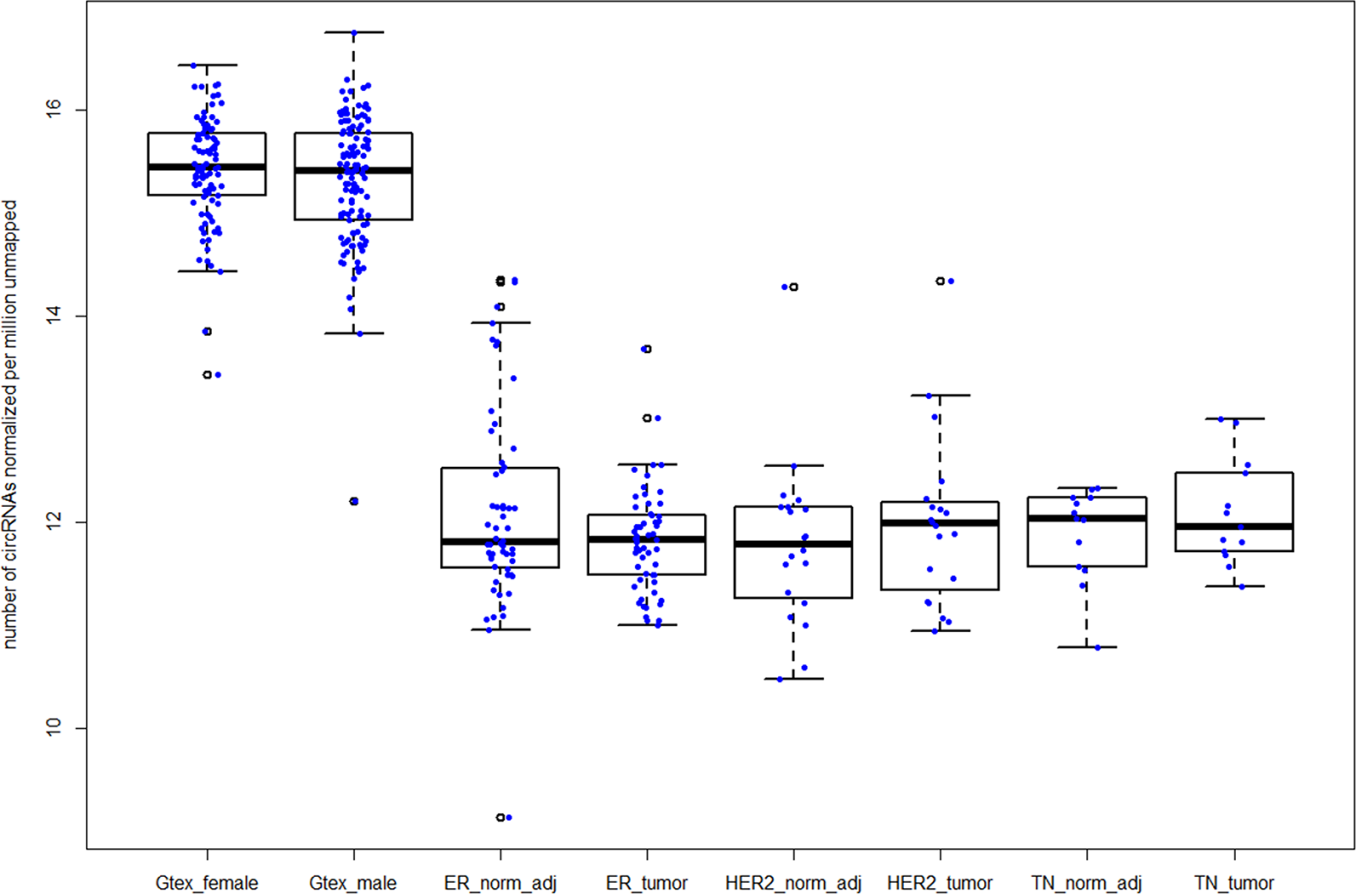
Increased number of circRNAs in normal breast samples compared to breast tumor subtypes in TCGA Legend from left to right – Gtex_female and Gtex_male represent female and male mammary tissues from the Gtex project; ER+, HER2+ and TN normal-adjacent and matched tumors from TCGA are represented by ER_norm_adj, ER_tumor, HER2_norm_adj, HER2_tumor, TN_norm_adj and TN_tumor respectively.

To confirm that number of circRNAs observed in normal samples is higher than breast tumors, we analyzed an independent cohort of 218 normal breast mammary tissues from the GTEx project (http://www.gtexportal.org/home/). After normalizing for library size, we observed higher number of circRNAs compared all three TCGA breast subtypes (Figure 4). Circ-Seq results for Gtex samples are provided in Supplementary File S6.

### circRNAs are negatively correlated with tumor proliferation markers in ER+ breast cancers

Recently, Bachmayr-Heyda et al. [22] reported that total number of circRNAs is negatively correlated with tumor proliferation marker MKI67 in colorectal cancer. Here we used a collection of 11 genes: *BIRC5, CCNB1, CDC20, CEP55, MKI67, NDC80, NUF2, PTTG1, RRM2, TYMS,* and *UBE2C* that are signatures for proliferation and are also part of the PAM50 classification gene panel [30]. We calculated the risk-of-relapse proliferation score (ROR-P) [24] for these genes to see if they have similar negative correlations with breast cancer subtypes.

**Figure 5:**
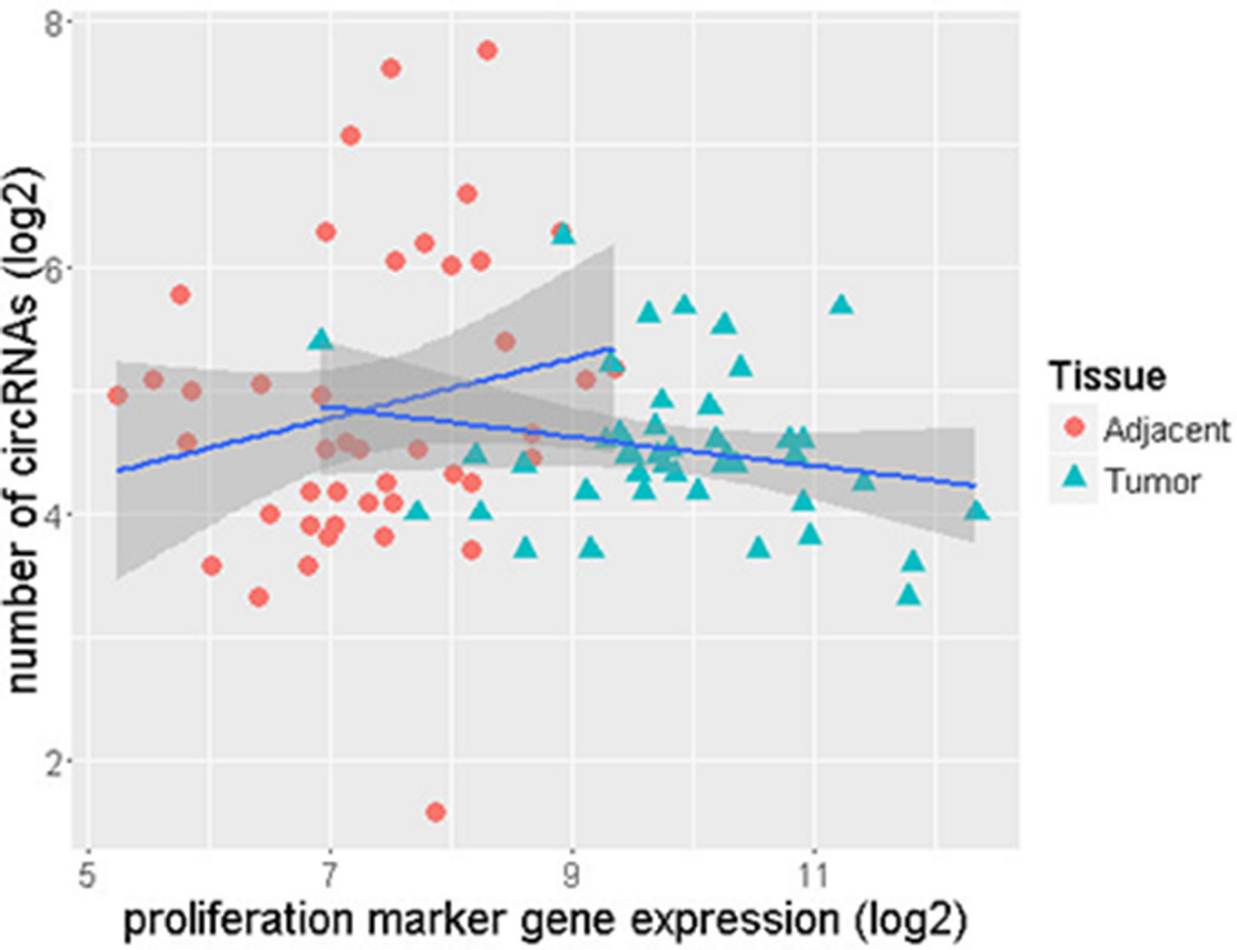
Lower number of circRNAs as gene proliferation increases in ER+ tumor samples

We observed that ER+ normal-adjacent tissue samples had a higher number of circRNAs and displayed lower levels of proliferation marker gene expression than ER+ tumor samples. Figure 5 is plotted between the ROR-P score and circRNA numbers for the tumor samples and indicates that the number of circRNAs in the ER+ tumors tends to decrease with average increase in gene proliferation. This trend is explained by a slightly negative correlation of −0.22. However, a corresponding analysis of paired HER2+ and TN samples revealed positive correlations −0.15 in HER2+ and 0.24 in TN tumors (Supplementary Figure S1A and S1B).

### ER+ luminal A and luminal B tumor tissues have distinct proliferation patterns based on number of circRNAs

**Figure 6:**
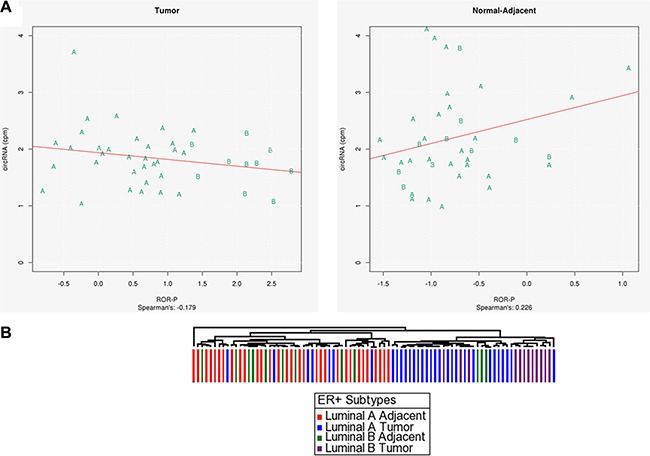
(**A**) Luminal A and Luminal b tumor samples show distinct separation based on their circRNA numbers when plotted against tumor proliferation, (**B**) Unsupervised hierarchical clustering analysis shows separation of Luminal A and Luminal B tumor and adjacent samples based on their circRNA numbers.

Because circRNAs appear to be promising markers for proliferation in ER+ tumors, and since number of circRNAs were significantly different between normal-adjacent and tumors, (*p*-value < 0.027), we further investigated if they could distinguish between the luminal A and luminal B types, as luminal B tumors proliferate more rapidly. First, we used PAM50 centroid modeling to identify tumor and normal-adjacent Luminal A and B subtypes for TCGA ER+ samples. Next, using all matched tumor and normal-adjacent ER+ samples (56 pairs), we plotted the number of circRNAs with respect to tumor proliferation. A clear distinction between the two ER+ types was evident for the tumor samples (Figure 6A). Luminal B tumors had fewer circRNAs (18 on average) than Luminal A tumors (25 on average) and this difference in circRNAs number was significant with *p*-value < 0.011 using Welch *t*-test. Luminal B normal–adjacent samples had similar number of circRNAs to luminal A normal–adjacent samples –24 and 30 on average, respectively, which was not statistically significant (*p* value = 0.31) (Supplementary Figure S2). An unsupervised hierarchical clustering analysis, shown in Figure 6B, also indicated that tumor and normal-adjacent samples cluster separately based on their circRNA numbers. In addition, Luminal B tumors separated out into their own sub-cluster within the tumor arm. These results suggest that Luminal A and B tumor samples show distinct differences in terms of proliferation marker gene expression based on their circRNA numbers. We hypothesize that this measure may be of use for other cancers with heterogeneous subtypes.

## DISCUSSION

In this study, using existing bioinformatics approaches defined by Memczak et al. [12] we developed a comprehensive analytical workflow called Circ-Seq. We also introduced three essential filters for identification and characterization of stable and expressed circRNAs in Circ-Seq. The workflow was designed with flexibility to allow users to configure these filters based upon their choice to report results that are either stringent or lenient. Circ-Seq is also designed with speed in mind. It is built to work on a multi-threaded cluster environment and can analyze numerous samples in parallel at any given time.

Circ-Seq was applied to the transcriptome of 885 TCGA breast cancer samples and we identified numerous circRNAs unique to breast tumors and normal-adjacent tissues. To our knowledge this is the first report to catalogue circRNAs unique to the TN, HER2+, and ER+ molecular subtypes of breast cancer, as well as circRNAs common to all of the subtypes but absent from normal-adjacent tissue. Finally, using a panel of 11 tumor proliferation marker genes in combination with circRNA abundance, we show that circRNA number is associated with tumor proliferation and that luminal A and luminal B tumors have distinct representations of circRNA numbers within the ER+ breast molecular subtype.

We also identified circRNAs in the breast cell lines and were able to successfully validate the largest circRNA identified in MCF7 found at genomic location chr14:102,466,325–102,500,789 with 12 supporting junction reads. This circRNA was a spliced product of gene *DYNC1H1* and spanned from exons 17 to 56 of the gene. Considering that the exon-intron structure remains intact, the size of this circRNA is about 7 kb and may play a role in post-transcriptional regulation. Notably, the circRNA contained microRNA response elements (MRE) for miR-150 and miR-661 with 29 and 23 unique binding sites respectively. These two microRNAs have been previously reported to have associations with cancer [31, 32]. In searching for other microRNAs that have over 20 binding sites, we found non-conserved microRNAs such as miR-3613, miR-4731 and miR-5095, each contain 25 MRE sites along the circRNA. It is possible that since the circRNA contains several binding sites for microRNAs, this could be a candidate player in breast cancer competing endogenous RNA (ceRNA) networks.

Recent studies suggest that circRNAs have other functions that are more common than the microRNA sponge effect. Notably, circRNAs are shown to participate actively with pre-mRNA splicing [10] and also as active promoters of transcription of parent mRNAs [17]. We believe that the circRNAs reported in this study can also have implications similar to the above functions in breast cancer.

Although validation results suggests that the workflow reports legitimate circRNAs, the reliability of the workflow and the measure of the false positive rate can only be determined based on its application to more transcriptome datasets and validation of results in future. The number of unmapped reads is a key player in identifying circRNAs within a sample. We observed that unmapped reads for the breast tumor and non-tumor cell lines range was 5–22 million and the range for TCGA samples was 5–78 million. Samples with unmapped reads at the low end of the spectrum can likely have correspondingly low number of circRNAs reported. We hypothesize that the number of circRNAs identified for BT20, T47D, MCF7, and MCF10A were artificially low due to the small number of unmapped reads available for these samples.

One of the limitations of this study is that the RNA-Seq libraries from TCGA are prepared using Illumina TruSeq, that enriches for poly-A tail transcripts [33], thus greatly limiting the number of circRNAs detected. Despite this limitation we identified large numbers of circRNAs in the TCGA breast cancer data. Stranded total RNA and RiboMinus libraries may improve the detection of circRNAs [2, 3, 12, 13, 15, 18]. We acknowledge that the circRNAs identified here are only a small subset of the actual population of circRNAs present in breast cancer samples. Because the number of circRNAs detected increases with the number of samples investigated, as shown in Table 2, the number of circRNAs detected for the TN and HER2+ subtypes is probably underestimated due to their smaller sample size. This could also be indicative of why we observed poor correlations and non-significant probability measures for these subtypes when the corresponding associations always held true for ER+ samples. Likewise, it is uncertain at this point whether the tumor proliferation analysis for TN and HER2+ patients with matched tumor and normal-adjacent tissues would indeed have negative correlation with circRNA numbers or not, if adequate number of samples were available for these subtypes.

Taking together the biological complexities in cancer, individual RNA classes cannot be considered in isolation. Cooperative communication between different types of noncoding RNAs and protein-coding genes or messenger RNAs exists [34–37] which eventually tune the expression of target genes. In cancer, regulated expression of tumor suppressors and oncogenes is critical to tumorigenesis. Competing endogenous RNA networks comprising of complex interactions between messenger RNA, microRNA and circRNA molecules can greatly influence the post-transcriptional activity of such genes. Messenger RNA stability, or lack of stability—depending on how the circRNAs and microRNAs interact via microRNA binding sites—can significantly impact gene expression, with serious repercussions for tumorigenesis. Innovative and ingenious bioinformatics techniques need to be developed that can unravel ceRNA crosstalk between such RNA types and eventually lead to novel findings which can be used as potential diagnostic targets to improve treatment of cancer. It is possible that the findings that emerge from the study of circRNAs will lead to improvements in the diagnosis and treatment of complex, heterogeneous diseases such as cancer.

## MATERIALS AND METHODS

### Circ-Seq workflow

**Figure 7:**
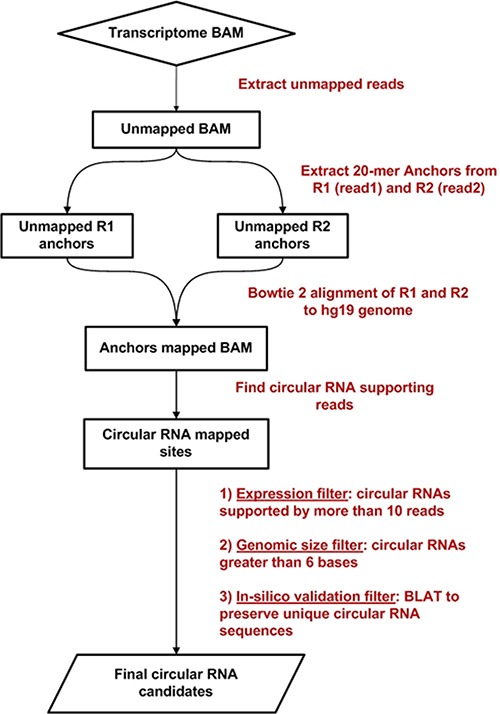
Circ-Seq bioinformatics workflow flowchart

The Circ-Seq workflow flowchart is represented in Figure 7. Circ-Seq is an extension of the circRNA detection methodology by Memczak *et al.* [12] and incorporates essential filters as well as comprehensive annotation to the final list of circRNA candidates. Circ-Seq starts by fragmenting unmapped reads from the aligned transcriptome BAM file into short 20-mer anchors from their 5′ and 3′ ends and are then realigned against the reference genome. For every unmapped read, if the anchor pair maps in a 3′ to 5′ fashion, the alignment is shortlisted as possible evidence for a circRNA. Next, acceptor and donor splice sites, i.e., AG and GT, are checked for the selected 3′ and 5′ anchors. The presence of anchors within the splice sites is treated as initial confirmation of the fusion of exons in a circRNA fashion. At this point, the workflow quantifies the number of anchors supporting each circRNA candidate.

Next, three unique filters are applied to eliminate unexpressed and false-positive circRNAs: an expression filter, a genomic size filter, and a validation filter. The expression filter retains circRNA candidates supported by a sufficient number of junction-spanning reads and is set to 5 reads by default, which is considerably more stringent that existing approaches [12].The genomic size filter discards any candidates shorter than 6 bases. Finally, to ensure that circRNAs reported by the workflow are not identified from repetitive regions of the genome, the validation filter uses BLAT [25] to confirm that the 3′ (head) and 5′ (tail) coordinates of the circRNA represent unique locations of the genome. After completing the analysis, the workflow provides a circRNA quantification report and a FASTA file that contains 50-base nucleotide sequences containing the 3′–5′ fused junction of all circRNAs identified.

### TCGA breast cancer transcriptome data

We downloaded 1,034 breast cancer RNA-Seq binary alignment map (BAM) files from the TCGA Research Network (http://cancergenome.nih.gov/) using the National Cancer Institute (NCI) Genomic Data Commons (GDC) resource (https://gdc.cancer.gov/). The un-stranded Illumina TrueSeq protocol was used to obtain 50 nucleotide paired-end reads from TCGA breast cancer RNA-Seq samples. The paired-end reads were then aligned using MapSplice v12_07 [34]; these reads contained both reads mapped to the human reference genome (hg19 / NCBI 37.1) and unmapped reads.

### TCGA breast tumor and normal-adjacent samples and normal breast mammary tissue from GTEx

We obtained clinical metadata for the 1,034 breast cancer samples from the NCI GDC Data Portal (https://gdc-portal.nci.nih.gov/). Because TCGA continues to add breast cancer cases to its repository, the most recent number of breast cancer samples available from TCGA may be higher than the number used in this work. We first classified the samples into the three predominant molecular subtypes – TN, ER+ and HER2. Out of 1,034 samples, we were able to classify subtypes for 885 samples of which 561 were ER+, 141 were TN, and 183 were HER2+ samples (141 + 183 + 561 = 885). Details on clinical classification of the 885 samples are provided in Supplementary Materials.

We downloaded BAM files for 218 normal breast samples (126 male and 92 female samples) from the Gtex project (http://www.gtexportal.org/home/) using Aspera client (http://asperasoft.com/). Samples were sequenced using Illumina TrueSeq paired-end RNA sequencing with read length 75 bp. The transcriptome BAM files downloaded for the 218 samples were aligned to the hg19 reference genome using Tophat [38].

### Breast cancer cell lines

We also obtained RNA-Seq paired-end sequence files for six breast cancer cell lines (BT20, BT474, MCF7, MDAMB468, T47D, and ZR751) and one cell line derived from normal breast cells (MCF10A) [26]. Sequences from the cell lines were processed using the Mayo Analysis Pipeline for RNA Sequencing (MAP-RSeq) to yield BAM files for use with the Circ-Seq workflow [39]. The number of unmapped reads for the cell lines varied from 5 to 22 million reads.

### Pathway analysis for tumor-specific circRNAs

Gene names and annotations of those that either overlap or neighbor tumor-specific circRNAs were obtained using the Ensembl reference system (version GRCh37.75). Enriched canonical pathway analysis for tumor-specific circRNAs in the breast molecular subtypes was performed using the Ingenuity pathway analysis software IPA (Ingenuity^®^ Systems, www.ingenuity.com). Biological functions and diseases information within the IPA software was used for critical investigation of cancer-related pathways. Open source analysis toolkit WebGestalt [29] was also used to derive pathway results (Supplementary File S7).

### CircRNA validation

MCF7 breast cancer cells (American Type Culture Collection Manassas, VA) were cultured in EMEM medium containing 10% fetal bovine serum (FBS) at 37°C in 5% CO_2_. Total RNA and genomic DNA were isolated using the RNeasy Plus Micro Kit and DNeasy Blood & Tissue Kit (QIAGEN, Inc.,Valencia, CA) respectively. DNA and RNA quality was analyzed using the NanoDrop 8000 spectrophotometer. qRT-PCR was performed with the Power SYBR^®^ Green RNA-to-CTTM 1-Step Kit (AB, Foster, CA) using a Stratagene Mx3005P Real-Time PCR detection system. *GAPDH* DNA and RNA were used as controls for the experiment. We designed two sets of primers, convergent primers that bound to linear 5′–3′ mRNA transcripts and divergent primers that bound to the circRNA transcript (chr14:102,466,325–102,500,789) formed in a 3′–5′ fashion (Supplementary Table S2), which were provided by Integrated DNA Technologies. After gel purification using the QIAquick Gel Extraction Kit (QIAGEN), the qRT-PCR product was sequenced using the Sanger method to confirm the head-to-tail splicing.

## SUPPLEMENTARY MATERIALS

















## References

[1] Guo JU, Agarwal V, Guo H, Bartel DP (2014). Expanded identification and characterization of mammalian circular RNAs. Genome Biol.

[2] Salzman J, Gawad C, Wang PL, Lacayo N, Brown PO (2012). Circular RNAs are the predominant transcript isoform from hundreds of human genes in diverse cell types. PLoS One.

[3] Jeck WR, Sorrentino JA, Wang K, Slevin MK, Burd CE, Liu J, Marzluff WF, Sharpless NE (2013). Circular RNAs are abundant, conserved, and associated with ALU repeats. Rna.

[4] Suzuki H, Zuo Y, Wang J, Zhang MQ, Malhotra A, Mayeda A (2006). Characterization of RNase R-digested cellular RNA source that consists of lariat and circular RNAs from pre-mRNA splicing. Nucleic Acids Res.

[5] Sanger HL, Klotz G, Riesner D, Gross HJ, Kleinschmidt AK (1976). Viroids are single-stranded covalently closed circular RNA molecules existing as highly base-paired rod-like structures. Proc Natl Acad Sci USA.

[6] Diener TO (1979). Viroids: structure and function. Science.

[7] Capel B, Swain A, Nicolis S, Hacker A, Walter M, Koopman P, Goodfellow P, Lovell-Badge R (1993). Circular transcripts of the testis-determining gene Sry in adult mouse testis. Cell.

[8] Pasman Z, Been MD, Garcia-Blanco MA (1996). Exon circularization in mammalian nuclear extracts. Rna.

[9] Chao CW, Chan DC, Kuo A, Leder P (1998). The mouse formin (Fmn) gene: abundant circular RNA transcripts and gene-targeted deletion analysis. Mol Med.

[10] Ashwal-Fluss R, Meyer M, Pamudurti NR, Ivanov A, Bartok O, Hanan M, Evantal N, Memczak S, Rajewsky N, Kadener S (2014). circRNA biogenesis competes with pre-mRNA splicing. Mol Cell.

[11] Wang PL, Bao Y, Yee MC, Barrett SP, Hogan GJ, Olsen MN, Dinneny JR, Brown PO, Salzman J (2014). Circular RNA is expressed across the eukaryotic tree of life. PLoS One.

[12] Memczak S, Jens M, Elefsinioti A, Torti F, Krueger J, Rybak A, Maier L, Mackowiak SD, Gregersen LH, Munschauer M, Loewer A, Ziebold U, Landthaler M (2013). Circular RNAs are a large class of animal RNAs with regulatory potency. Nature.

[13] Salzman J, Chen RE, Olsen MN, Wang PL, Brown PO (2013). Cell-type specific features of circular RNA expression. PLoS Genet.

[14] Jeck WR, Sharpless NE (2014). Detecting and characterizing circular RNAs. Nat Biotechnol.

[15] Lukiw WJ (2013). Circular RNA (circRNA) in Alzheimer's disease (AD). Front Genet.

[16] Valdmanis PN, Kay MA (2013). The expanding repertoire of circular RNAs. Mol Ther.

[17] Li Z, Huang C, Bao C, Chen L, Lin M, Wang X, Zhong G, Yu B, Hu W, Dai L, Zhu P, Chang Z, Wu Q (2015). Exon-intron circular RNAs regulate transcription in the nucleus. Nat Struct Mol Biol.

[18] Hansen TB, Jensen TI, Clausen BH, Bramsen JB, Finsen B, Damgaard CK, Kjems J (2013). Natural RNA circles function as efficient microRNA sponges. Nature.

[19] Bahn JH, Zhang Q, Li F, Chan TM, Lin X, Kim Y, Wong DT, Xiao X (2015). The landscape of microRNA, Piwi-interacting RNA, and circular RNA in human saliva. Clin Chem.

[20] Li Y, Zheng Q, Bao C, Li S, Guo W, Zhao J, Chen D, Gu J, He X, Huang S (2015). Circular RNA is enriched and stable in exosomes: a promising biomarker for cancer diagnosis. Cell Res.

[21] Yang JH, Li JH, Shao P, Zhou H, Chen YQ, Qu LH (2011). starBase: a database for exploring microRNA-mRNA interaction maps from Argonaute CLIP-Seq and Degradome-Seq data. Nucleic Acids Res.

[22] Bachmayr-Heyda A, Reiner AT, Auer K, Sukhbaatar N, Aust S, Bachleitner-Hofmann T, Mesteri I, Grunt TW, Zeillinger R, Pils D (2015). Correlation of circular RNA abundance with proliferation—exemplified with colorectal and ovarian cancer, idiopathic lung fibrosis, and normal human tissues. Sci Rep.

[23] Ghosal S, Das S, Sen R, Basak P, Chakrabarti J (2013). Circ2Traits: a comprehensive database for circular RNA potentially associated with disease and traits. Front Genet.

[24] Nielsen TO, Parker JS, Leung S, Voduc D, Ebbert M, Vickery T, Davies SR, Snider J, Stijleman IJ, Reed J, Cheang MC, Mardis ER, Perou CM (2010). A comparison of PAM50 intrinsic subtyping with immunohistochemistry and clinical prognostic factors in tamoxifen-treated estrogen receptor-positive breast cancer. Clin Cancer Res.

[25] Kent WJ (2002). BLAT—the BLAST-like alignment tool. Genome Res.

[26] Sun Z, Asmann YW, Kalari KR, Bot B, Eckel-Passow JE, Baker TR, Carr JM, Khrebtukova I, Luo S, Zhang L, Schroth GP, Perez EA, Thompson EA (2011). Integrated analysis of gene expression, CpG island methylation, and gene copy number in breast cancer cells by deep sequencing. PLoS One.

[27] Dai X, Li Y, Bai Z, Tang X-Q (2015). Molecular portraits revealing the heterogeneity of breast tumor subtypes defined using immunohistochemistry markers. Sci Rep.

[28] Pouladi N, Cowper-Sallari R, Moore JH (2014). Combining functional genomics strategies identifies modular heterogeneity of breast cancer intrinsic subtypes. BioData Min.

[29] Zhang B, Kirov S, Snoddy J (2005). WebGestalt: an integrated system for exploring gene sets in various biological contexts. Nucleic Acids Res.

[30] Martin M, Prat A, Rodriguez-Lescure A, Caballero R, Ebbert MT, Munarriz B, Ruiz-Borrego M, Bastien RR, Crespo C, Davis C, Rodriguez CA, Lopez-Vega JM, Furio V (2013). PAM50 proliferation score as a predictor of weekly paclitaxel benefit in breast cancer. Breast Cancer Res Treat.

[31] Aherne ST, Madden SF, Hughes DJ, Pardini B, Naccarati A, Levy M, Vodicka P, Neary P, Dowling P, Clynes M (2015). Circulating miRNAs miR-34a and miR-150 associated with colorectal cancer progression. BMC Cancer.

[32] Hoffman Y, Bublik DR, Pilpel Y, Oren M (2014). miR-661 downregulates both Mdm2 and Mdm4 to activate p53. Cell Death Differ.

[33] Illumina (2010). Illumina TrueSeq RNA preperation.

[34] Prueitt RL, Yi M, Hudson RS, Wallace TA, Howe TM, Yfantis HG, Lee DH, Stephens RM, Liu CG, Calin GA, Croce CM, Ambs S (2008). Expression of microRNAs and protein-coding genes associated with perineural invasion in prostate cancer. Prostate.

[35] Schetter AJ, Leung SY, Sohn JJ, Zanetti KA, Bowman ED, Yanaihara N, Yuen ST, Chan TL, Kwong DL, Au GK, Liu CG, Calin GA, Croce CM (2008). MicroRNA expression profiles associated with prognosis and therapeutic outcome in colon adenocarcinoma. Jama.

[36] Farazi TA, Horlings HM, Ten Hoeve JJ, Mihailovic A, Halfwerk H, Morozov P, Brown M, Hafner M, Reyal F, van Kouwenhove M, Kreike B, Sie D, Hovestadt V (2011). MicroRNA sequence and expression analysis in breast tumors by deep sequencing. Cancer Res.

[37] Creighton CJ, Hernandez-Herrera A, Jacobsen A, Levine DA, Mankoo P, Schultz N, Du Y, Zhang Y, Larsson E, Sheridan R, Xiao W, Spellman PT, Getz G (2012). Integrated analyses of microRNAs demonstrate their widespread influence on gene expression in high-grade serous ovarian carcinoma. PLoS One.

[38] Trapnell C, Pachter L, Salzberg SL (2009). TopHat: discovering splice junctions with RNA-Seq. Bioinformatics.

[39] Kalari KR, Nair AA, Bhavsar JD, O'Brien DR, Davila JI, Bockol MA, Nie J, Tang X, Baheti S, Doughty JB, Middha S, Sicotte H, Thompson AE (2014). MAP-RSeq: Mayo Analysis Pipeline for RNA sequencing. BMC Bioinformatics.

