# Supplementary material for: Circular RNAs and their associations with breast cancer subtypes
Source: Oncotarget. 2016 Nov 5;7(49):80967–79. doi: 10.18632/oncotarget.13134 (PMC5348369; doi:10.18632/oncotarget.13134)
Supplement: Supplementary file 1 [file oncotarget-07-80967-s001.pdf]

Circular RNAs and their associations with breast cancer subtypes

Supplementary Materials

Recurrent circRNAs have higher expression levels in tumors compared to normal-adjacent tissue

We identified unique circRNAs that are recurrent in tumor and normal-adjacent tissues by classifying the TCGA breast samples into three categories – low (25%), medium (75%) and high (100%). At low frequency range, circRNAs are identified in a single or few samples whereas at high frequency, circRNAs are highly recurrent among samples. As shown in Supplementary Table S1, the number of unique circRNAs increased as the level of frequency detection depreciates for both tumor and normal-adjacent tissues. At high range of sample frequency, TN has 11 tumor and 14 normal-adjacent circRNAs, ER+ with 8 and 12, and HER2+ has 9 and 16 tumor and normal-adjacent circRNAs respectively that are recurrent. Interestingly, the average expression (number of junction supporting reads) for these recurrent circRNAs are significantly higher in tumor (p-value < 0.055) when compared to the paired normal-adjacent tissues.

circRNA validation in MCF7 cell line

Using Circ-Seq, we identified 9 circRNAs in the MCF7 breast tumor cell line. We chose the circRNA with the largest genomic size (7 kb) for validation. *GAPDH* DNA and RNA were used as controls for the experiment. We designed two sets of primers, convergent primers that bound to linear 5′–3′ mRNA transcripts and divergent primers that bound to the circRNA transcript (chr14:102,466,325–102,500,789) formed in a 3′–5′ fashion. The primers were provided by Integrated DNA Technologies. Supplementary Table S2 provides the

Supplementary Table S1: The number of circRNAs identified in matched tumor and adjacent tissue samples, stratified by cancer type (triple negative, estrogen receptor+, or HER2+) and sample frequency (low, medium, or high)

Frequency of samples	Number of samples in frequency range	Number of unique circRNAs	Average junction supporting reads	Number of samples in frequency range	Number of unique circRNAs	Average junction supporting reads
**Triple Negative (TN)**						
		Tumor, n = 128			Normal-adjacent, n = 13	
25% (low)	32	1,224	5.1	3	106	6.7
75% (medium)	63	296	4.8	5	88	5
100% (high)	128	11	15.2	13	14	7.2
**Estrogen Receptor (ER+)**						
		Tumor, n = 503			Normal-adjacent, n = 56	
25% (low)	126	2,959	3.3	14	1,219	5.1
75% (medium)	251	388	3.8	26	184	2.5
100% (high)	503	8	11.6	56	12	8
**ERBB2 overexpressed (HER2+)**						
		Tumor, n = 162			Normal-adjacent, n = 20	
25% (low)	40	2,452	4.4	5	173	5.8
75% (medium)	81	343	3.8	8	96	5.1
100% (high)	162	9	18	20	16	9.3

forward and reverse primer sequences for convergent and divergent primers used for validation.

Supplementary Table S2: Convergent and divergent primer sequences for validation of circRNA in the MCF7 cell line

	Primer Pair		Primer sequence (3′–5′)
GAPDH	Convergent	Forward	AGCAAGAGCACAAGAGGAAG
		Reverse	TGGTACATGACAAGGTGCG
	Divergent	Forward	TCCTCACAGTTGCCATGTAGACCC
		Reverse	TGCGGGCTCAATTTATAGAAACCGGG
circRNA on			
chr 14	Convergent	Forward	CACTTCAGGCTCTCACCATAT
		Reverse	AGCCCAGTATCTGTCAATTCC
	Divergent	Forward	GTACAGATATTGCCAGGACGG
		Reverse	TCCTTCAGCCTACCAAACTTC

Analysis of circRNA numbers for paired samples in HER2+ and TN samples

We obtained paired breast tumor and normal-adjacent tissue sequence data from 13 patients with TN cancer and 20 patients with HER2+ cancer. The number of unique circRNAs of each sample was compared against gene expression of 11 proliferation markers to check for correlation. Although circRNA results showed a higher number of unique circRNAs in normal-adjacent samples than matched tumors for 5 TN patients and 6 HER2+ patients, we did not observe a statistically significant correlation for these molecular subtypes. Using standard paired *t*-test calculation, we observed *p*-value = 0.73 for TN and *p*-value = 0.59 for HER2+ patients. Supplementary Figure S1 shows the linear regression line for both normal-adjacent and tumor samples of the TN and HER2+ subtypes.

Analysis of circRNA numbers for normal-adjacent samples of ER+ Luminal A and Luminal B subtypes

The ER+ paired samples (56 pairs) were further classified into Luminal A and Luminal B types using in-house PAM50 modeling techniques. While we observed a clear distinction between Luminal A and Luminal B tumor samples in terms of their circRNA numbers with respect to the gene expression of 11 proliferation markers, no such separation was seen for the normal-adjacent

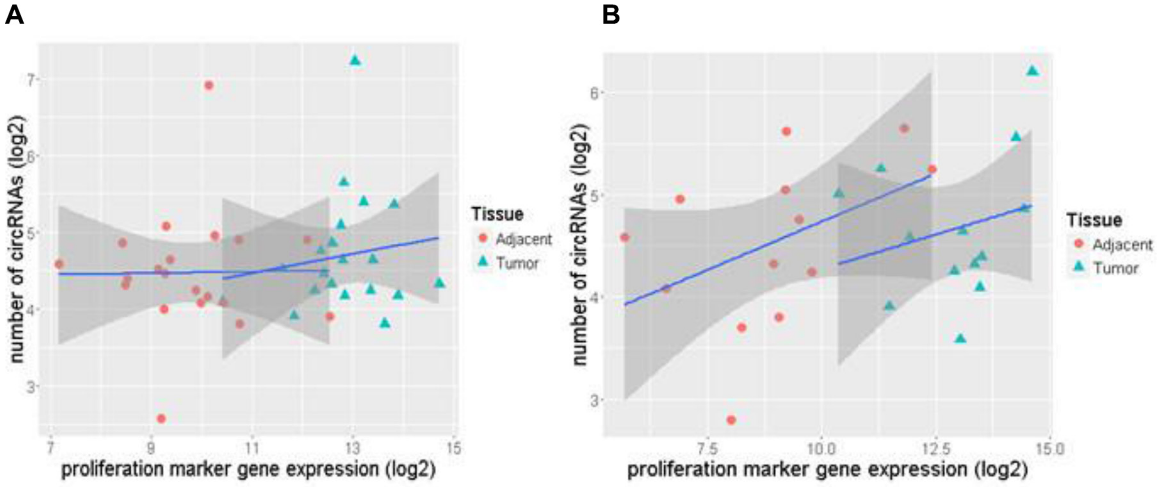

Supplementary Figure S1: No correlation between number of circRNAs and expression of proliferation marker genes was observed in (A) HER2+ and (B) triple negative (TN) paired tumor and adjacent tissue samples.

samples. As shown in Supplementary Figure 2, Luminal B normal–adjacent samples had similar number of circRNAs to luminal A normal–adjacent samples –24 and 30 on average, respectively, which was not statistically significant (p value = 0.31).

Using the molecular subtype classification, we were able to classify 885 of the 1,034 TCGA samples into their respective subtypes. The biospecimen metadata did not contain clinical annotation for 149 samples and thus were excluded from analysis. As shown in Supplementary Figure 3, of the 885 samples, 561 were ER+, 140 were TN, and 183 were HER2+. The ER+ samples were composed of 503 tumors, 56 adjacent tissue samples, and 2 metastatic samples. TN samples comprised 128 tumors, 12 adjacent samples, and no metastases. HER2+ samples included 162 tumors, 20 normal-adjacent samples, and 1 metastatic sample. All adjacent and metastatic tissue samples were matched to tumor samples from the same patient.

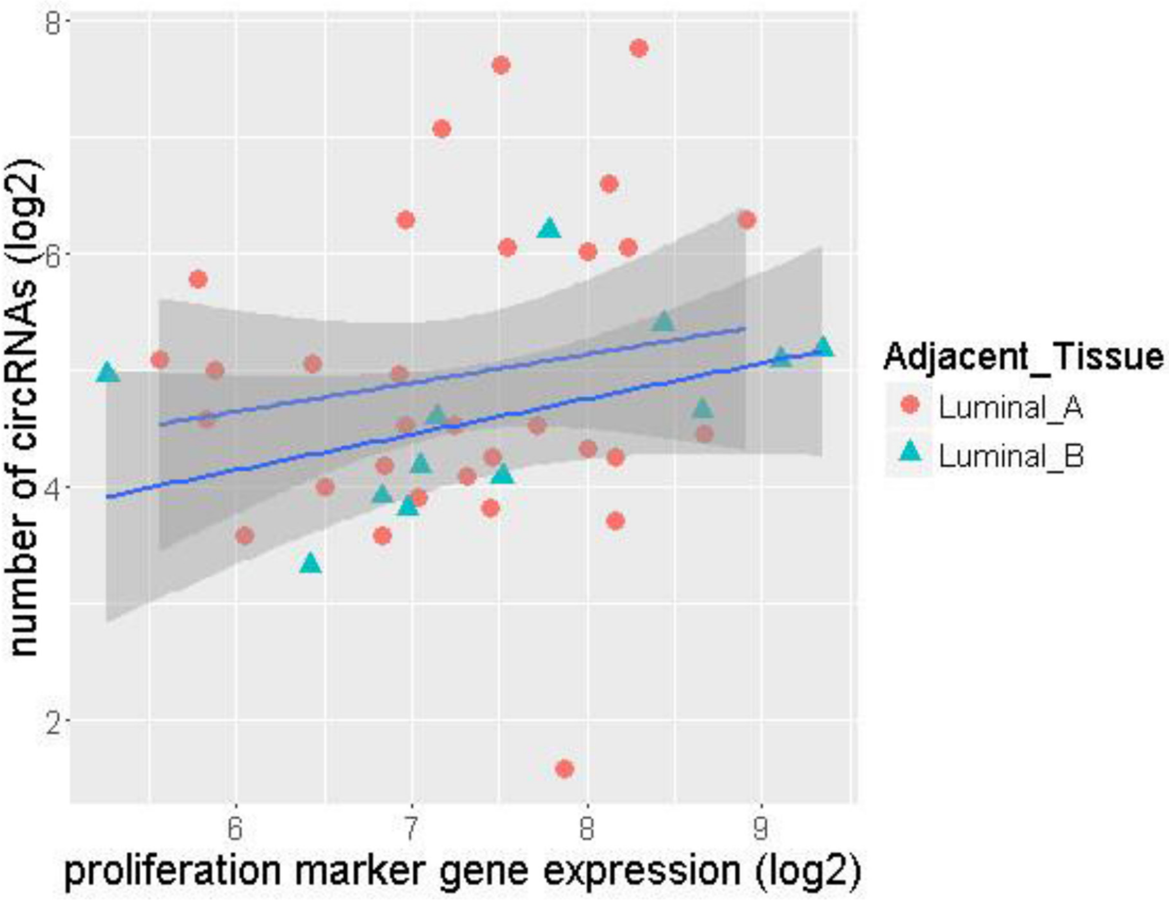

Supplementary Figure S2: Number of circular RNAs between Luminal A and Luminal B normal-adjacent samples have similar trends when their circRNA numbers are plotted against proliferation.

Clinical subtype classification of TCGA breast tumor and normal-adjacent samples

To classify the TCGA breast cancer samples, we chose two important parameters from the biospecimen metadata available from TCGA. The first parameter was the classification of samples based on the three predominant molecular subtypes in breast cancer: ER+, HER2+, and TN. The second parameter was the tissue status of the sample: primary tumor, normal-adjacent tissue, or metastasis.

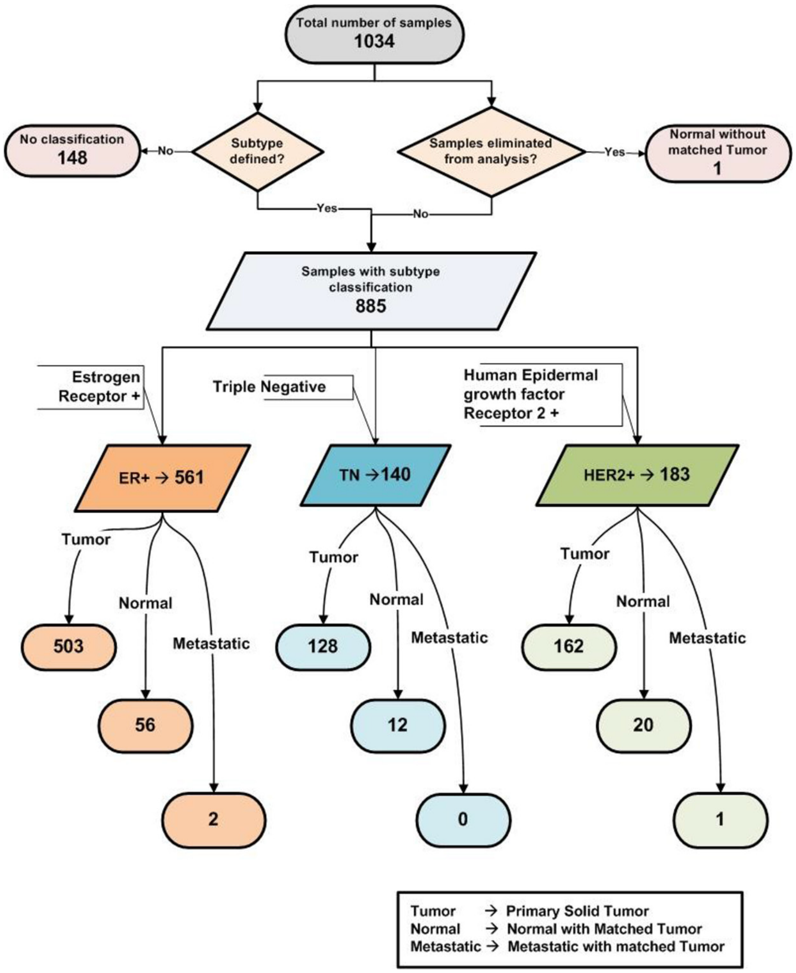

Supplementary Figure S3: TCGA breast cancer sample flowchart. Samples were first categorized based on their molecular subtype (estrogen receptor+, HER2+, or triple negative) followed by categorization based on tissue type (primary tumor, normal-adjacent tissue, or metastasis).

Supplementary File S1: circRNAs identified in tumor and normal breast cell lines. see Supplementary_File_S1.

Supplementary File S2: Tumor, normal-adjacent and tumor-specific circRNAs identified in Estrogen Receptor positive (ER+) TCGA breast samples. see Supplementary_File_S2.

Supplementary File S3: Tumor, normal-adjacent and tumor-specific circRNAs identified in Triple Negative (TN) TCGA breast samples. see Supplementary_File_S3.

Supplementary File S4: Tumor, normal-adjacent and tumor-specific circRNAs identified in ERBB2 overexpressed (HER2+) TCGA breast samples. see Supplementary_File_S4.

Supplementary File S5: Number of distinct circRNAs identified in tumor and normal-adjacent tissues for TN, ER+ and HER2+ paired samples. see Supplementary_File_S5.

Supplementary File S6: circRNAs identified in Gtex normal mammary tissues. see Supplementary_File_S6.

Supplementary File S7: Canonical pathways identified using the WebGestalt toolkit for tumor-specific circRNAs in TN, ER+ and HER2_ subtypes. see Supplementary_File_S7.
